# A unique arsenic speciation profile in *Elaphomyces* spp. (“deer truffles”)—trimethylarsine oxide and methylarsonous acid as significant arsenic compounds

**DOI:** 10.1007/s00216-018-0903-3

**Published:** 2018-02-12

**Authors:** Simone Braeuer, Jan Borovička, Walter Goessler

**Affiliations:** 1 Institute of Chemistry, Analytical Chemistry for Health and Environment, University of Graz, Universitaetsplatz 1, 8010 Graz, Austria; 2 The Czech Academy of Sciences, Nuclear Physics Institute, Hlavní 130, 25068 Husinec-Řež, Czech Republic; 3 The Czech Academy of Sciences, Institute of Geology, Rozvojová 269, 16500 Prague 6, Czech Republic

**Keywords:** *Elaphomyces*, Fungi, Deer truffles, Arsenic speciation, Trimethylarsine oxide, Methylarsonous acid

## Abstract

Arsenic and its species were investigated for the first time in nine collections of *Elaphomyces* spp. (“deer truffles”) from the Czech Republic with inductively coupled plasma mass spectrometry (ICPMS) and high-performance liquid chromatography coupled to ICPMS. The total arsenic concentrations ranged from 12 to 42 mg kg^−1^ dry mass in samples of *E. asperulus* and from 120 to 660 mg kg^−1^ dry mass in *E. granulatus* and *E. muricatus*. These concentrations are remarkably high for terrestrial organisms and demonstrate the arsenic-accumulating ability of these fungi. The dominating arsenic species in all samples was methylarsonic acid which accounted for more than 30% of the extractable arsenic. Arsenobetaine, dimethylarsinic acid, and inorganic arsenic were present as well, but only at trace concentrations. Surprisingly, we found high amounts of trimethylarsine oxide in all samples (0.32–28% of the extractable arsenic). Even more remarkable was that all but two samples contained significant amounts of the highly toxic trivalent arsenic compound methylarsonous acid (0.08–0.73% of the extractable arsenic). This is the first report of the occurrence of trimethylarsine oxide and methylarsonous acid at significant concentrations in a terrestrial organism. Our findings point out that there is still a lot to be understood about the biotransformation pathways of arsenic in the terrestrial environment.

**Electronic supplementary material:**

The online version of this article (10.1007/s00216-018-0903-3) contains supplementary material, which is available to authorized users.

**Graphical abstract:**
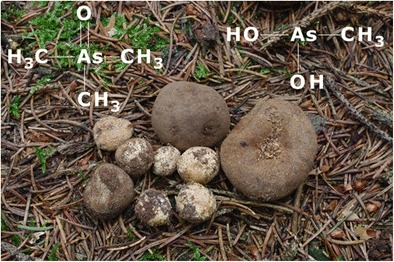
Trimethylarsine oxide and methylarsonous acid in “deer truffles”

## Introduction

Arsenic is occurring in the environment in many chemical forms. Distinguishing between these different compounds is essential, because arsenic’s toxicity strongly depends on the element’s species. In water, the main arsenic compounds are the two inorganic species arsenous acid [As (III)] and arsenic acid [As(V)] [1], which possess high acute and also chronic toxicity [2]. As (III) and As(V) are also the main arsenic species in soil extracts, using water (with or without small amounts of salts, for example phosphate) and/or methanol as extracting solution [3]. Additionally, small amounts of organic arsenic compounds such as methylarsonic acid (MA), dimethylarsinic acid (DMA), arsenobetaine (AB), or trimethylarsine oxide (TMAO) can sometimes also be found in soils [4]. The extractable arsenic from plants is in most cases predominantly inorganic arsenic [5], although there are also reports on MA and DMA as major arsenical in plant extracts [6, 7]. Significant amounts of other arsenicals like TMAO are found in plants only very rarely [8]. The main arsenic metabolites of terrestrial mammals are MA and DMA [9, 10]. In rare occasions, small amounts of the trivalent methylated arsenic species methylarsonous acid [MA(III)] and dimethylarsinous acid have also been detected in urine of terrestrial mammals [11]. Apart from urine, MA(III) has only been detected in a sample of soil that was treated with MA(V) [12] and in carrots with unusually high total arsenic concentrations [13]. Until now, MA(III) has not been found in any other environmental sample. The detection of this compound in feed or foodstuff would be alarming, because studies have shown that MA(III) is even more toxic than As(III) [14].

In the marine ecosystem, the arsenic concentrations and arsenic speciation are completely different from the terrestrial environment. First, the total arsenic concentrations in marine organisms are usually much higher than in terrestrial ones, and second, the major arsenic species in fish or algae are AB or more complex molecules like arsenosugars, or, more recently discovered, lipid-soluble arsenic species, which are seldom found in significant concentrations in terrestrial samples [15, 16]. One big exception is macrofungi. Depending on the fungal species, they can have similarly high concentrations of total arsenic as marine organisms (up to more than 1000 mg kg^−1^ dm) [17, 18]. Further, macrofungi are one of the few terrestrial organisms that can contain AB as main arsenic species [19, 20]. In other fungal species, inorganic arsenic and DMA are the dominating arsenic compounds [21, 22]. MA is most often only present at very low concentrations, or even not at all, and only rarely a major constituent of the fungal arsenic speciation [19]. Up to now, TMAO, arsenocholine (AC), the tetramethyl arsonium ion (TETRA), and arsenosugars have been found much more seldom in macrofungi, and mostly only at trace concentrations [22].

There are thousands of macrofungal species in temperate ecosystems, but the arsenic speciation has been investigated only in a very small part of it so far. For example, there is no information at all about arsenic compounds in hypogeous fungi (which produce macroscopic fruit-bodies partially or completely embedded in soil or humus, truffle-like fungi), and even data on the total arsenic concentrations in these organisms are scarce. To the best of our knowledge, there are only two publications on arsenic in hypogeous fungi; one is written by Orczán et al., who investigated 22 elements, including arsenic, in 17 different hypogeous fungi species, 93 samples in total [23]. They found on average 4 ± 12 mg As kg^−1^ dry mass (dm) in these samples (3 ± 10 mg As kg^−1^ dm when looking at *Elaphomyces* spp*.* only, *n* = 50). The second report is by Ljubojevic et al., who found 4.4 mg As kg^−1^ in soil and 2.1 mg As kg^−1^ (probably fresh mass) in *Choiromyces meandriformis* [24].

*Elaphomyces* (“deer truffles”) is one of the most important ectomycorrhizal fungal genera in temperate and subarctic forest ecosystems [25]. Preliminary neutron activation screening of arsenic in ascocarps (fruit-bodies) of several *Elaphomyces* species revealed elevated arsenic concentrations, up to hundreds of mg kg^−1^ dm. For this reason, we collected several ascocarps of three *Elaphomyces* species (*E. granulatus, E. muricatus*, and *E. asperulus*) and determined the concentrations of arsenic and around 30 other elements with inductively coupled plasma mass spectrometry (ICPMS) as well as their arsenic speciation with high-performance liquid chromatography (HPLC) coupled to ICPMS.

## Experimental

### Sample collection, identification, and preparation

*Elaphomyces* samples were collected in Bohemia, Czech Republic, at spruce plantations mostly from places dug by wild boars; determination of species is based on morphological characters. Ascocarps were thoroughly brushed in distilled water and frozen. Six samples (sample IDs: ASP-44, ASP-55, ASP-57, ASP-56, ASP-58, and ASP-59) were lyophilized. One sample of *E. granulatus* (ID: ASP-84) and two samples of *E. asperulus* (IDs: ASP-85a and ASP-85b) were kept frozen until analysis. They were thawed, homogenized with an ultra-centrifugal mill (ZM200, 1 mm titanium sieve, Retsch GmbH, Haan, Germany), digested, and also extracted within 1 day. The water content was determined in a drying oven at 100 °C for around 16 h.

### Determination of total element concentrations

All homogenized samples were digested in a microwave heated pressurized digestion system (Ultraclave 4, MLS GmbH, Leutkirch, Germany). Each sample was prepared in triplicates. First, about 100 mg (weighed to 0.1 mg) of the samples was put into quartz vessels. 5 mL nitric acid (≥ 65% m/m p.a., Carl Roth GmbH + Co.KG, Karlsruhe, Germany, further purified via sub-boiling) was added and the vessels were closed loosely with PTFE-caps. The digestion oven was loaded with 4.0*10^6^ Pa of argon (5.0, Messer, Gumpoldskirchen, Austria) and then heated up to 250 °C. The temperature was held for 30 min, and then the system was cooled down again. The digests were transferred to 50 mL PP-tubes (Greiner Bio-one, Kremsmünster, Austria) and diluted with ultrapure water (18.2 MΩ*cm, Merck Millipore, Bedford, USA) to a final volume of 50 mL (final concentration of nitric acid: 10% *v*/*v*). For quality control, the Standard Reference Materials® (SRM) 1573a (Tomato Leaves, NIST, Gaithersburg, USA, *n* = 15) and SRM® 1568b (Rice Flour, *n* = 5) were digested together with the samples as well as blanks (*n* = 22).

The element concentrations were determined with an inductively coupled plasma triple quadrupole mass spectrometer (ICPQQQMS, Agilent 8800, Agilent Technologies, Waldbronn, Germany). The instrument was equipped with a MicroMist nebulizer, a Scott-type spray chamber, and Cu/Ni cones. The following elements were analyzed in the samples: Ag, Al, As, B, Ba, Bi, Ca, Cd, Co, Cr, Cs, Cu, Fe, Gd, Hg, K, Li, Mg, Mn, Mo, Na, Ni, P, Pb, Rb, S, Sb, Se, Sn, Sr, Te, Tl, U, V, Zn. The selected collision/reaction cell modes (helium, hydrogen, oxygen and no cell gas) and mass to charge ratios and the settings of the instrument can be found in the Electronic Supplementary Material (ESM, Tables S1 and S2). Quantification was obtained via external calibration. The calibration solutions were prepared in 15 mL PP-tubes (Greiner Bio-one) and consisted of 10% *v*/*v* of nitric acid (≥ 65% m/m p.a., Carl Roth GmbH + Co.KG) and aliquots of the single element standards (Carl Roth GmbH + Co.KG). Each calibration standard contained all elements except mercury, which was prepared in separate solutions. These contained 8% *v*/*v* nitric acid and 2% *v*/*v* hydrochloric acid (Rotipuran® 37% m/m, p.a., subboiled twice in-house, Carl Roth GmbH + Co.KG) for a better stabilization of the element.

For quality assurance and quality control, the digested SRMs® 1573a and 1568b as well as SRM® 1640a (Trace Elements in Natural Water, diluted 1 + 9 with ultrapure water and 10% *v*/*v* nitric acid, *n* = 7) were measured with the samples. After every 10th sample, a calibration standard was re-measured to determine a possible drift of the instrument. 200 μg L^−1^ of Be, Ge, In, and Lu (1% *v*/*v* nitric acid) were added online via a t-piece in front of the nebulizer to all samples and served as internal standards.

### Arsenic speciation analysis

For extraction, 200 mg of the samples was weighed to 0.1 mg into 15 mL PP-tubes (Greiner Bio-one). Each sample was prepared in triplicates. Four mL of ultrapure water was added. The mixtures were shaken, put into an ultrasonic bath for 15 min (Transsonic T 700/H, Elma GmbH&Co.KG, Singen, Germany) and then centrifuged at 3300×*g* for 10 min (Rotina 420 R, Hettich Lab Technology, Tuttlingen, Germany). The extracts were filtered with syringes (Norm-Ject, Henke-Sass Wolf GmbH, Tuttlingen, Germany) through 0.2 μm polyamide syringe filters (Chromafil® Xtra PA-20/13, Macherey-Nagel GmbH & Co. KG, Düren, Germany). One part of one filtered replicate of each sample was mixed with 10% *v*/*v* of hydrogen peroxide (Rotipuran®, 30% m/m p.a., stabilized, Carl Roth GmbH + Co.KG), and put into an oven for 1 h at 45 °C.

Arsenic speciation analysis was carried out with HPLC-ICPQQQMS, on the same day of extraction. The HPLC system consisted of an Agilent 1200 HPLC, equipped with a degasser, a quaternary pump, a thermostatted autosampler, and a thermostatted column compartment. We applied anion-exchange chromatography for the determination of As(V), DMA, MA and MA(III), and cation-exchange chromatography for the determination of AB, TMAO, AC, and TETRA. The methods have been validated elsewhere [26]. For anion-exchange chromatography, a PRP-X100 column (150 * 4.6 mm, 5 μm, Hamilton, Bonaduz, Switzerland) and an aqueous phosphate buffer (20 mM, pH 6.0, adjusted with ammonia, 1 mL min^−1^, 40 °C) were used. A Zorbax 300-SCX column (150 * 4.6 mm, 5 μm, Agilent) and an aqueous pyridine solution (10 mM, pH 2.3, adjusted with nitric acid, 1.5 mL min^−1^, 30 °C) were employed for cation-exchange chromatography. The injection volume was 20 μL in both methods. Ammonium dihydrogen phosphate (Suprapur 99.99%), ammonia solution (Suprapur 25% m/m), and pyridine (30% m/m p.a.) were obtained from Merck KGaA (Darmstadt, Germany).

The arsenic signal was detected with ICPQQQMS in oxygen reaction mode at m/z 91 (^75^As^+^ ➔ ^75^As^16^O^+^). 15% *v*/*v* CO_2_ (1% *v*/*v* in Ar) was added as optional gas between spray chamber and torch to enhance the arsenic signal and compensate for any possible carbon enhancement effect from the organic matrix of the extracts.

Quantification and identification of the arsenic species were achieved via external calibration (0.05–100 μg As L^−1^ for each compound). The calibration solutions were prepared in 15 mL PP-tubes (Greiner Bio-one) with ultrapure water and aliquots of standard solutions of the different arsenic species. These standard solutions (1000 mg As L^−1^ each) were prepared as follows. As [V] was prepared from Na_2_HAsO_4_*7 H_2_O, purchased from Merck (Darmstadt, Germany). Methylarsonic acid (MA) was synthesized from NaAsO_2_ (purchased from Merck) and MeI (Meyer reaction). DMA was prepared from sodium dimethylarsinate (Fluka, Buchs, Switzerland). MA(III) was prepared by dissolving methyl arsenic diiodide in water with 5% *v*/*v* methanol. AB, TMAO, AC and TETRA were synthesized according to literature [27–30].

Due to the instability of MA(III) was not added to the calibration standards, but was quantified via the calibration of DMA. Its identity was checked by spiking the extract in the following manner: 20 μL of the extract and 2 μL of a solution of MA(III) with a 10 times higher concentration than in the extract were taken up by the injector needle and then injected together onto the column. Additionally, the disappearance of the peak after the addition of hydrogen peroxide confirmed the initial presence of MA(III).

During anion- and also during cation-exchange chromatography, one calibration standard was re-measured after every 10th sample for stability control. Since there is no certified reference material for arsenic species in a matrix that is comparable to mushrooms, we injected SRM® 1640a (Trace elements in natural water, *n* = 3) and compared the inorganic arsenic concentration with the certified value for total arsenic.

The extraction efficiency was determined by diluting all extracts with 1% *v*/*v* nitric acid and then measuring the arsenic signal with ICPQQQMS (m/z 75 ➔91, oxygen mode, plus 15% v/v CO_2_ as optional gas). Quantification was obtained with external calibration.

The identity of TMAO in the extracts was verified via HPLC- electrospray ionization mass spectrometry (ES-MS, 6120, Agilent Technologies). Again, the cation-exchange column Zorbax 300-SCX was employed with 0.5 M formic acid and 0.03 M ammonium formate (pH = 2.3) and 8% *v*/*v* methanol as mobile phase. The flow rate was 1.5 mL min^−1^, and the flow was split via a T-piece after the column; one part was going to the ES-MS, and one to the waste. The injection volume was 1 μL. The settings of the ES-MS were 90 V fragmentor voltage, 1000 V capillary voltage, 350 °C gas temperature, 12 L min^−1^ drying gas. TMAO was recorded in the SIM mode at a *m*/*z* ratio 137 ((CH_3_)_3_AsOH^+^).

## Results

The results for all reference materials were generally in good agreement with the certified values, as can be found in ESM Table S3.

The water content of the three fresh samples was between 49 and 52%. In order to be able to compare the results with the dried samples, the individual water content values were used to convert the results of the fresh samples into concentrations on a dry mass basis.

The total arsenic concentrations in the samples ranged from 12 to 660 mg kg^−1^ dm. The three samples of *E. asperulus* contained between 12 and 42 mg As kg^−1^ dm, whereas the arsenic concentration in the four samples of *E. granulatus* ranged from 120 to 660 mg kg^−1^ dm. The two samples of *E. muricatus* contained 180 ± 30 and 280 ± 10 mg kg^−1^ dm. Interestingly, the concentrations of Na, K, Rb and, less pronounced, Cs were lower in *E. asperulus* than in the other samples. For example, *E. asperulus* contained only 37–310 mg Na kg^−1^ dm, whereas the other samples contained 2800–4700 mg Na kg^−1^ dm. The concentrations of arsenic and the alkali metals are listed in Table 1. All other elements can be found in ESM Table S4.

**Table 1:** Concentrations of total arsenic (mg kg^−1^ dm), extracted arsenic (mg kg^−1^ dm and % of the total arsenic in brackets), sum of all arsenic species (mg kg^−1^ dm and % of the extracted arsenic in brackets), arsenic species (mg kg^−1^ dm), and the alkali elements (mg kg^−1^ dm) in the investigated samples of *Elaphomyces*

Sample ID	ASP-44	ASP-55	ASP-57	ASP-84	ASP-56	ASP-58	ASP-59	ASP-85a	ASP-85b
Species	*E. granulatus*	*E. granulatus*	*E. granulatus*	*E. granulatus*	*E. muricatus*	*E. muricatus*	*E. asperulus*	*E. asperulus*	*E. asperulus*
State when analyzed	Dried	Dried	Dried	Fresh	Dried	Dried	Dried	Fresh	Fresh
Total As	151 ± 8	400 ± 30	660 ± 30	120 ± 7	180 ± 30	280 ± 10	12 ± 1	18 ± 1	42 ± 1
Extracted As	130 ± 10 (83 ± 9%)	330 ± 3 (81.6 ± 0.7%)	514 ± 8 (77 ± 1%)	110 ± 20 (90 ± 10%)	145 ± 1 (79.6 ± 0.8%)	150 ± 3 (54 ± 1%)	1.8 ± 0.2 (14 ± 2%)	1.1 ± 0.1 (6.3 ± 0.8%)	1.3 ± 0.09 (3.1 ± 0.2%)
Sum of species	130 ± 20 (99 ± 2%)	280 ± 40 (85 ± 10%)	440 ± 40 (85 ± 9%)	94 ± 5 (90 ± 20%)	132 ± 5 (91 ± 4%)	136 ± 6 (91 ± 5%)	1.4 ± 0.3 (80 ± 10%)	0.9 ± 0.2 (80 ± 20%)	0.80 ± 0.1 (65 ± 10%)
MA	100 ± 10	280 ± 30	420 ± 40	93 ± 6	130 ± 5	94 ± 3	0.86 ± 0.09	0.7 ± 0.2	0.48 ± 0.05
TMAO	22 ± 3	2.1 ± 0.3	9.9 ± 0.7	0.34 ± 0.03	0.7 ± 0.2	42 ± 3	0.5 ± 0.2	0.15 ± 0.03	0.31 ± 0.04
MA (III)	0.9 ± 0.2	1.2 ± 0.2	0.72 ± 0.09	0.9 ± 0.3	0.62 ± 0.08	0.22 ± 0.05	~ 0.01	< 0.002	< 0.002
DMA	~ 0.05	~ 0.03	0.2 ± 0.1	< 0.002	~ 0.03	0.21 ± 0.02	~ 0.02	~ 0.01	~ 0.01
AB	0.16 ± 0.04	~ 0.05	~ 0.06	< 0.002	~ 0.06	0.2 ± 0.1	~ 0.008	~ 0.003	~ 0.004
As (V)	~ 0.02	~ 0.05	0.09 ± 0.03	~ 0.02	~ 0.05	~ 0.04	~ 0.03	~ 0.004	~ 0.003
Unkown species (sum)	~ 0.1	~ 0.4	~ 0.7	~ 0.04	~ 0.2	~ 0.6	~ 0.004	< 0.002	~ 0.01
Li	0.019 ± 0.005	0.056 ± 0.002	0.082 ± 0.001	0.078 ± 0.005	0.021 ± 0.003	0.0454 ± 0.0009	0.01 ± 0.001	0.019 ± 0.001	0.048 ± 0.004
Na	3600 ± 200	4100 ± 400	3200 ± 100	3600 ± 200	2800 ± 200	4700 ± 300	310 ± 20	37 ± 2	60 ± 20
K	8200 ± 100	15,000 ± 2000	24,800 ± 700	16,100 ± 900	16,000 ± 1000	3900 ± 300	2500 ± 200	1380 ± 70	950 ± 60
Rb	611 ± 8	340 ± 20	820 ± 50	177 ± 9	580 ± 30	510 ± 30	108 ± 4	19 ± 1	13.9 ± 0.8
Cs	42.3 ± 0.8	73 ± 3	104 ± 1	14.6 ± 0.5	21.5 ± 0.7	51.5 ± 0.6	10.5 ± 0.2	3.5 ± 0.2	3.3 ± 0.1

The extraction efficiencies were 83 ± 6% for *E. granulatus*, around 80 and 54% for the two samples of *E. muricatus*, and only 3–14% for *E. asperulus.* The mean column recovery over all samples was 85 ± 10% (range 65–99%). Taken together with the extraction efficiency, this means that we were able to detect and quantify with arsenic speciation analysis only 2–11% of the total arsenic in *E. asperulus* and 49–82% in the other samples.

**Fig. 1:**
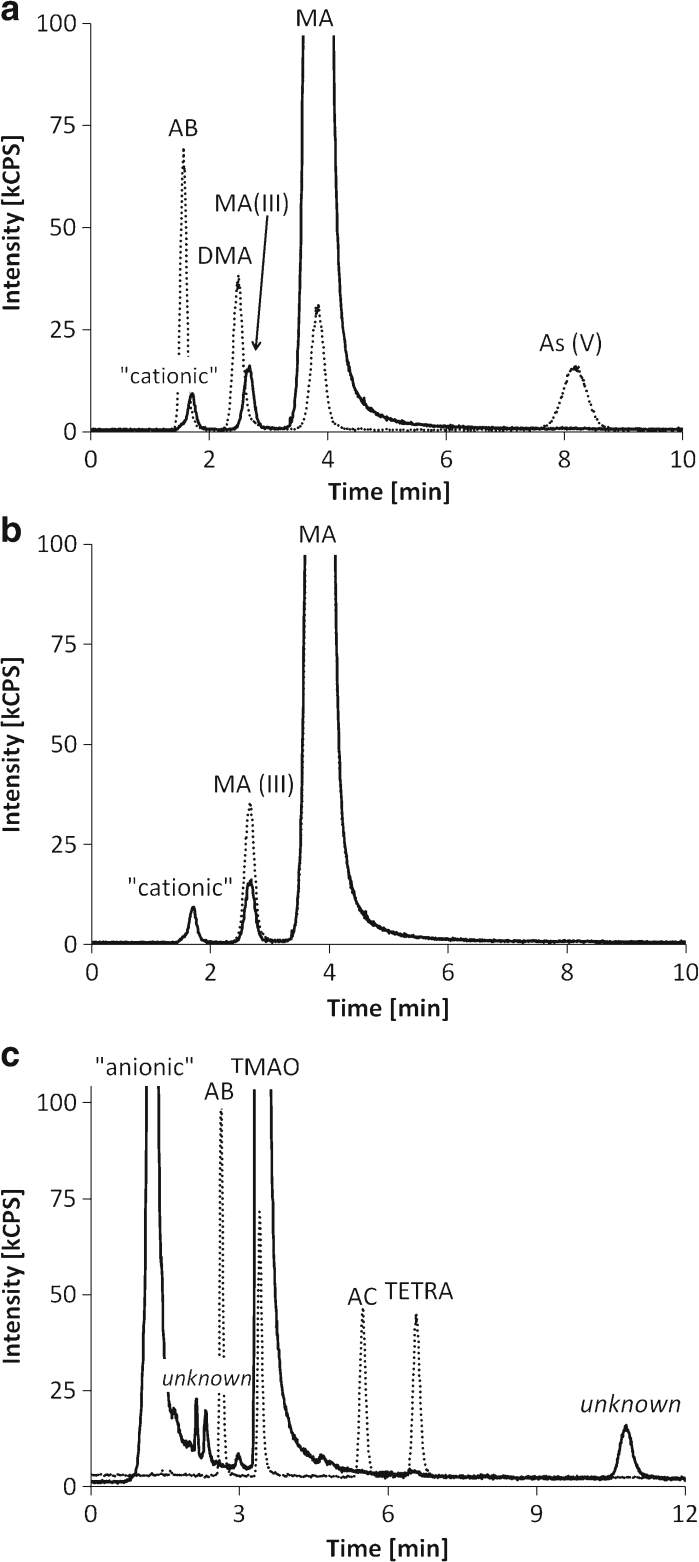
**a** Anion-exchange chromatograms of an extract (solid line) and of a standard, containing 5 μg As L^−1^ of AB, DMA, MA, and As(V) (dotted line). **b** Anion-exchange chromatograms of an extract; pure (solid line) and spiked with MA(III) (dotted line). **c** Cation-exchange chromatograms of an extract (solid line) and of a standard, containing 5 μg As L^−1^ of AB, TMAO, AC, and TETRA (dotted line)

The major arsenic species in all samples was MA, which accounted for 80 ± 20% of the sum of all arsenic species that were detected with HPLC-ICPMS. The second most abundant arsenic compound was TMAO, ranging from 0.37 to 37%, with a median of 17%. In absolute concentrations, this means 0.15–40 mg kg^−1^ dm. The identity of TMAO in the extracts was confirmed by HPLC-ES-MS; the chromatogram is provided in ESM Fig. S1. Inorganic arsenic accounted for 1–3.5% of the arsenic species in *E. asperulus* and only for around 0.01% of the arsenic species in the other samples. DMA and AB were generally only present at trace concentrations. Surprisingly, there were significant amounts of MA(III) in the extracts of *E. granulatus* and *E. muricatus*, confirmed by spiking and oxidation experiments (as described in the experimental section). This compound accounted for 0.16–0.74% of the detected arsenic species, which corresponds to up to 1.2 mg As kg^−1^ dm (see Fig. 1). Concerning *E. asperulus*, the compound was only detected in one of the samples (around 0.01% of the arsenic species). We also found small amounts of some unknown arsenic species (in total less than 1% of the arsenic species). One of these compounds was even eluting after around 11 min on the cation-exchange column (see Fig. 1c), which is very late compared to the most strongly retained known arsenic species, TETRA, with 6.5 min. Overall, there were no apparent differences between the fresh and the dried samples.

## Discussion

The total arsenic concentrations in our samples were quite high, namely up to 660 mg kg^−1^ dm. When compared to other macrofungi, our results are not on top of the arsenic accumulating species, but certainly in the upper part of the ranking [17]. The only two other publications on arsenic in hypogeous fungi that we are aware of found less than 10 mg As kg ^−1^ [23, 24], which is almost 100 times lower than the total arsenic concentrations in our samples of *E. granulatus* and *E. muricatus*. Even the three samples of *E. asperulus,* which contained 12–42 mg kg^−1^ dm, were higher than these literature values. Only the two samples with the highest arsenic concentrations (400 and 660 mg kg^−1^ dm) originated from mining areas with probably elevated arsenic concentrations in soil, while all other samples came from pristine regions and still contained up to 280 mg kg^−1^ dm. To find the reason for this discrepancy with the two other studies [23, 24], certainly more samples will have to be investigated.

Perhaps the most striking discovery of our study is the presence of significant amounts of MA(III) in most of the extracts. This compound has never been found in mushrooms before, and also the reports of MA(III) in other samples are very rare [11–13]. It has to be noted that the correct detection and quantification of this molecule has proven to be very tricky, because of its lability and quick oxidation to the pentavalent equivalent [31, 32]. On the other hand, in the case of the detection of MA(III) in carrots [13], there is a slight possibility that small amounts of the originally present pentavalent MA were reduced to MA(III) during extraction at elevated temperatures (60 °C). Of course, one cannot exclude to 100% that this also applies to our investigated *Elaphomyces* samples, but using pure water at room temperature as extraction agent was specifically chosen to influence the original arsenic speciation as little as possible.

Because of the quick oxidation of MA(III), it is possible that the concentration of MA(III) in our investigated samples is even underestimated. Regardless of the actual original concentrations of MA(III) in the samples, its pure presence is a unique discovery in the field of arsenic speciation in the environment.

Further, the dominating arsenic species in all extracts was MA, which has already been reported for a few other fungi, like *Sarcosphaera coronaria* [19], which is also an ascomycete. However, in most macrofungi, MA is only a minor arsenic compound or even not present at all [22]. The second most abundant arsenic compound in our samples was TMAO, accounting for up to 37% of the sum of arsenic species that were detected with HPLC-ICPMS or, in other words, up to 15% of the total arsenic.

Already in 1945, Challenger proposed a transformation pathway of inorganic arsenic by the filamentous fungus *Scopulariopsis brevicaulis* with consecutive reduction and methylation steps, via the penta- and trivalent forms of MA and DMA to TMAO and further to trimethylarsine (TMA) [33]. For humans and other terrestrial mammals, this is not directly applicable, because TMAO is hardly ever found in mammals’ urine. Hence, alternate mechanisms have been proposed, with DMA as final product [34]. Many macrofungi also contain DMA as main arsenic species, but in our investigated extracts of *Elaphomyces* spp., this compound is only present at trace concentrations. Instead, MA and TMAO make up for more than 90% of the speciated arsenic. One can speculate that the transformation of arsenic in these fungi could actually be quite close to the pathway described by Challenger. DMA would only be an intermediate that is quickly further methylated to TMAO and/or TMA. The latter one is actually volatile and has a very distinct smell. One could speculate that the hypogeous fungi are actively producing TMA (via TMAO) to attract wild boars and other mycophagous mammals. On the other hand, the ingestion of *Elaphomyces* spp*.* might pose an increased health risk for the animals, because MA(III) is highly toxic [14].

It has to be noted that there is no clear evidence that arsenic is transformed by macrofungi. Alternatively, associated microbiota could be responsible for the formation of the different arsenic compounds, which may be subsequently taken up by the fungi, but this theory is not proven either.

Interestingly, the extraction efficiencies were acceptable, though not 100%, for *E. granulatus* and *E. muricatus*, but less than 15% for *E. asperulus*. This means that there is still a large part of the fungal arsenic of which we do not know the chemical form. Since this arsenic is not extractable with water, one possibility could be lipid-soluble arsenicals. Another option would be that the arsenic is strongly attached or bound to large bio-molecules, such as proteins. Additional extraction experiments will be necessary to elucidate this question in the future.

## Conclusion

The investigated species of *Elaphomyces* are not only accumulating arsenic, but also possess a unique arsenic speciation; the two major arsenic compounds in the extracts were MA and TMAO and also significant amounts of MA(III) were detected. This is indicating that the arsenic metabolism of these organisms is very different from all other organisms that have been investigated so far. The reason for this is not clear at all. One very speculative hypothesis is that TMAO is further metabolized to TMA, which is then used for attracting mycophagous mammals. On the other hand, the presence of MA(III) might be a health risk for wild animals that feed on these mushrooms. Overall, our investigations show that definitely more work is needed to elucidate the role of arsenic in the terrestrial environment and its interactions with macrofungi.

## Electronic supplementary material

ESM 1 (PDF 1.05 mb)

ESM 2 (XLSX 12 kb)

ESM 3 (XLSX 13 kb)
