# Supplementary material for: A unique arsenic speciation profile in Elaphomyces spp. (“deer truffles”)—trimethylarsine oxide and methylarsonous acid as significant arsenic compounds
Source: Anal Bioanal Chem. 2018 Feb 12;410(9):2283–90. doi: 10.1007/s00216-018-0903-3 (PMC5849658; doi:10.1007/s00216-018-0903-3)
Supplement: Supplementary file 1 — (PDF 1.05 mb) [file 216_2018_903_MOESM1_ESM.pdf]

**Analytical and Bioanalytical Chemistry**

**Electronic Supplementary Material**

# A unique arsenic speciation profile in *Elaphomyces* spp. (“deer truffles”) – trimethylarsine oxide and methylarsonous acid as significant arsenic compounds

Simone Braeuer, Jan Borovička, Walter Goessler

Additional files available under 10.1007/s00216-018-0903-3.

**Table S1** All measured elements with the elected octopole gas modes and m/z ratios

| Element Name | octopole gas mode | m/z |
|---|---|---|
| Li | nogas | 7 |
| B | nogas | 11 |
| Na | He | 23 |
| Mg | He | 24 |
| Al | nogas | 27 |
| P | O2 | 31 -> 47 |
| S | O2 | 32 -> 48 |
| K | He | 39 |
| Ca | He | 43 |
| V | He | 51 |
| Cr | He | 52 |
| Mn | He | 55 |
| Fe | He | 56 |
| Co | He | 59 |
| Ni | He | 60 |
| Cu | He | 65 |
| Zn | He | 66 |
| As | O2 | 75 -> 91 |
| Se | H2 | 78 |
| Rb | He | 85 |
| Sr | He | 88 |
| Mo | nogas | 98 |
| Ag | nogas | 107 |
| Cd | nogas | 111 |
| Sn | nogas | 118 |
| Sb | nogas | 121 |
| Te | nogas | 125 |
| Cs | he | 133 |
| Ba | nogas | 137 |
| Gd | nogas | 157 |
| Hg | nogas | 201 |
| Tl | nogas | 205 |
| Pb | nogas | 208 |
| Bi | nogas | 209 |
| U | nogas | 238 |

**Table S2** Configuration of ICPQQQMS. Differing settings during HPLC-ICPMS in $O_2$-mode in brackets

| | O2 | H2 | He | nogas |
|---|---|---|---|---|
| Scan Type | MS/MS | Single Quad | Single Quad | Single Quad |
| RF Power [W] | 1600 | 1600 | 1600 | 1600 |
| RF Matching [V] | 1.8 | 1.8 | 1.8 | 1.8 |
| Smpl Depth [mm] | 8 | 8 | 8 | 8 |
| Carrier Gas [L/min] | 1.1 (0.85) | 1.1 | 1.1 | 1.1 |
| Option Gas [%] | 0 (15) | 0 | 0 | 0 |
| Nebulizer Pump [rps] | 0.1 (0.5) | 0.1 | 0.1 | 0.1 |
| S/C Temp [°C] | 2 | 2 | 2 | 2 |
| Extract 1 [V] | 0 | 0 | -1.5 | -1.5 |
| Extract 2 [V] | -160 | -150 | -195 | -185 |
| Omega Bias [V] | -90 | -80 | -110 | -95 |
| Omega Lens [V] | 6.6 | 6.6 | 7.9 | 7.9 |
| Q1 Entrance [V] | -1 | -1 | -3 | 0 |
| Q1 Exit [V] | -1 | -1 | -1 | -1 |
| Cell Focus [V] | 2 | -4 | -3 | -6 |
| Cell Entrance [V] | -50 | -50 | -50 | -50 |
| Cell Exit [V] | -60 | -60 | -60 | -60 |
| Deflect [V] | 3.2 | -60 | -6 | 13 |
| Plate Bias [V] | -60 | -60 | -60 | -60 |
| Q1 Bias [V] | -2 | -4 | -4 | -3 |
| Q1 Prefilter Bias [V] | -44 | -36 | -48 | -40 |
| Q1 Postfilter Bias [V] | -18 | -18 | -34 | -2 |
| He flow [mL/min] | 0 | 0 | 4 | 0 |
| H2 flow [mL/min] | 0 | 5.5 | 0 | 0 |
| 4th cell gas flow [%] | 25 | 0 | 0 | 0 |
| OcP Bias [V] | -5 | -18 | -18 | -8 |
| OcP RF [V] | 190 | 200 | 130 | 120 |
| Energy discrimination [V] | -7 | 0 | 3 | 5 |

**Table S3** and **Table S4** available under „Supplementary material“

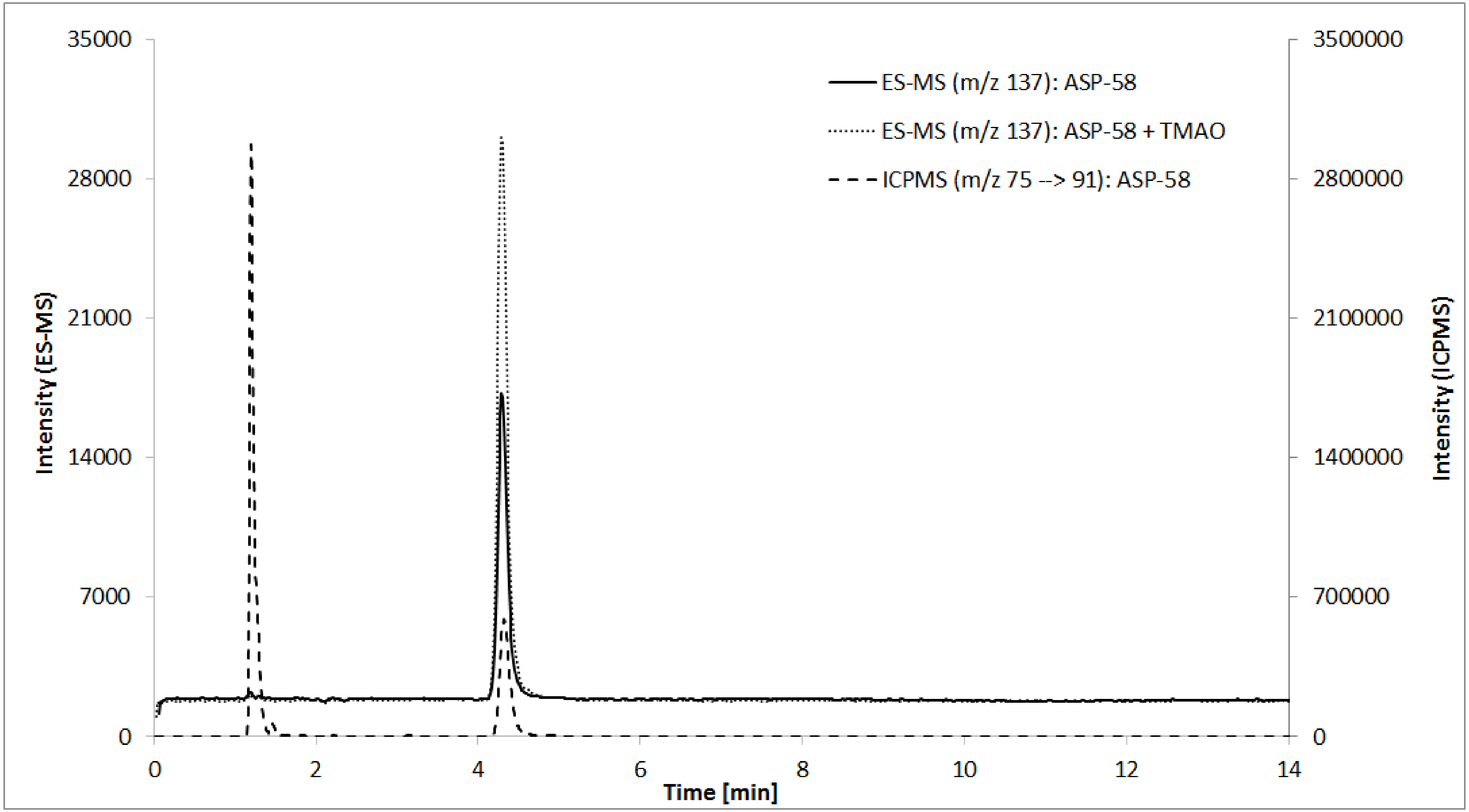


**Fig. S1** HPLC-ES-MS chromatograms of m/z 137 ($(CH_3)_3AsOH^+$). Solid line = extract of ASP-058, diluted 1+9 with water. Dotted line = same sample as for the solid line, spiked with a pure solution of TMAO (200 µg As $L^{-1}$). Dashed line = same sample and chromatographic method as for the solid line, but with ICPMS (ICPQQQMS 8800, oxygen mode, m/z 75 → 91) as detector instead of ES-MS
